# Supplementary material for: Splice-Junction-Based Mapping of Alternative Isoforms in the Human Proteome
Source: Cell Rep. Author manuscript; Available in PMC 2020 Jan 15. (PMC6961840; doi:10.1016/j.celrep.2019.11.026)
Supplement: 2 [file NIHMS1546469-supplement-2.zip › DF1/PXD010154/Thyroid/Thyroid_24_PDXDC1_QEVEATAGLLYVDDPNWSGIGVVSFRNSGR.pdf]

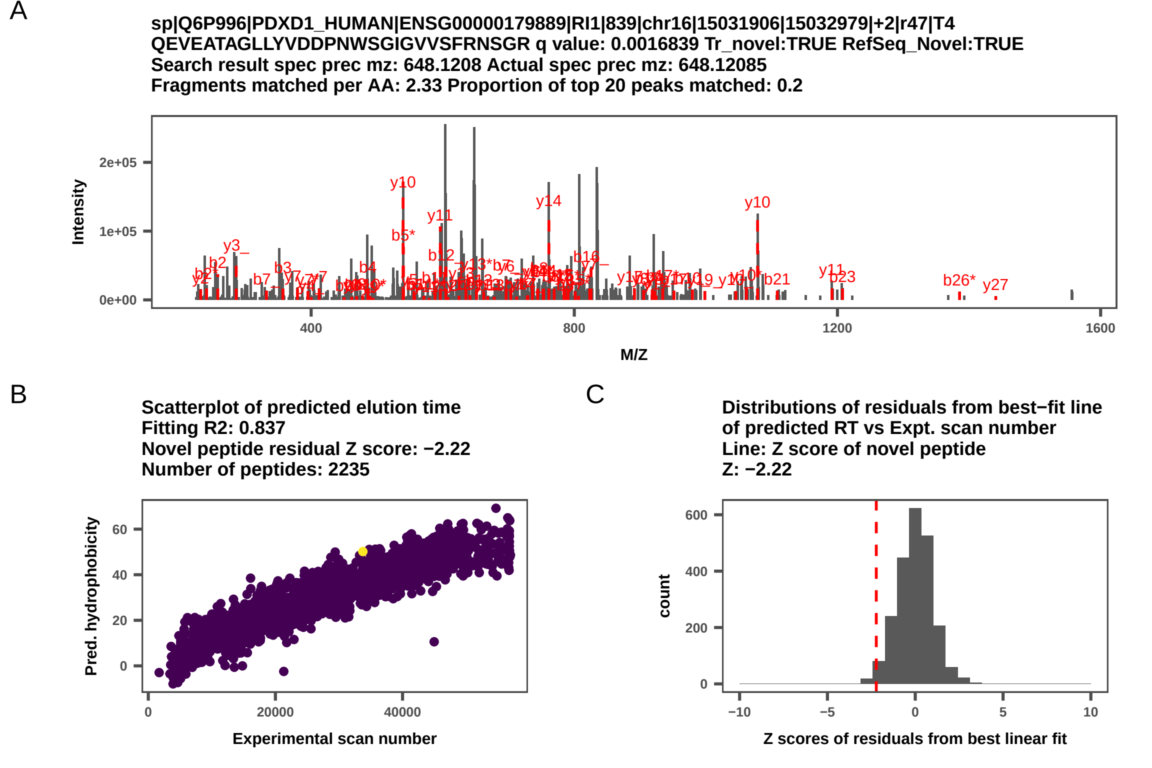
A
sp|Q6P996|PDXD1_HUMAN|ENSG00000179889|RI1|839|chr16|15031906|15032979|+2|r47|T4
QEVEATAGLLYVDDPNWSGIGVVSFRNSGR q value: 0.0016839 Tr_novel:TRUE RefSeq_Novel:TRUE
Search result spec prec mz: 648.1208 Actual spec prec mz: 648.12085
Fragments matched per AA: 2.33 Proportion of top 20 peaks matched: 0.2
Intensity
2e+05
1e+05
0e+00
400
800
1200
1600
M/Z
B
Scatterplot of predicted elution time
Fitting R2: 0.837
Novel peptide residual Z score: −2.22
Number of peptides: 2235
Pred. hydrophobicity
60
40
20
0
20000
40000
Experimental scan number
C
Distributions of residuals from best−fit line
of predicted RT vs Expt. scan number
Line: Z score of novel peptide
Z: −2.22
count
600
400
200
−10
−5
5
10
Z scores of residuals from best linear fit
